# Supplementary figures and images for: Chickpea Roots Undergoing Colonisation by Phytophthora medicaginis Exhibit Opposing Jasmonic Acid and Salicylic Acid Accumulation and Signalling Profiles to Leaf Hemibiotrophic Models
Source: Microorganisms. 2022 Feb 2;10(2):343. doi: 10.3390/microorganisms10020343 (PMC8874544; doi:10.3390/microorganisms10020343)

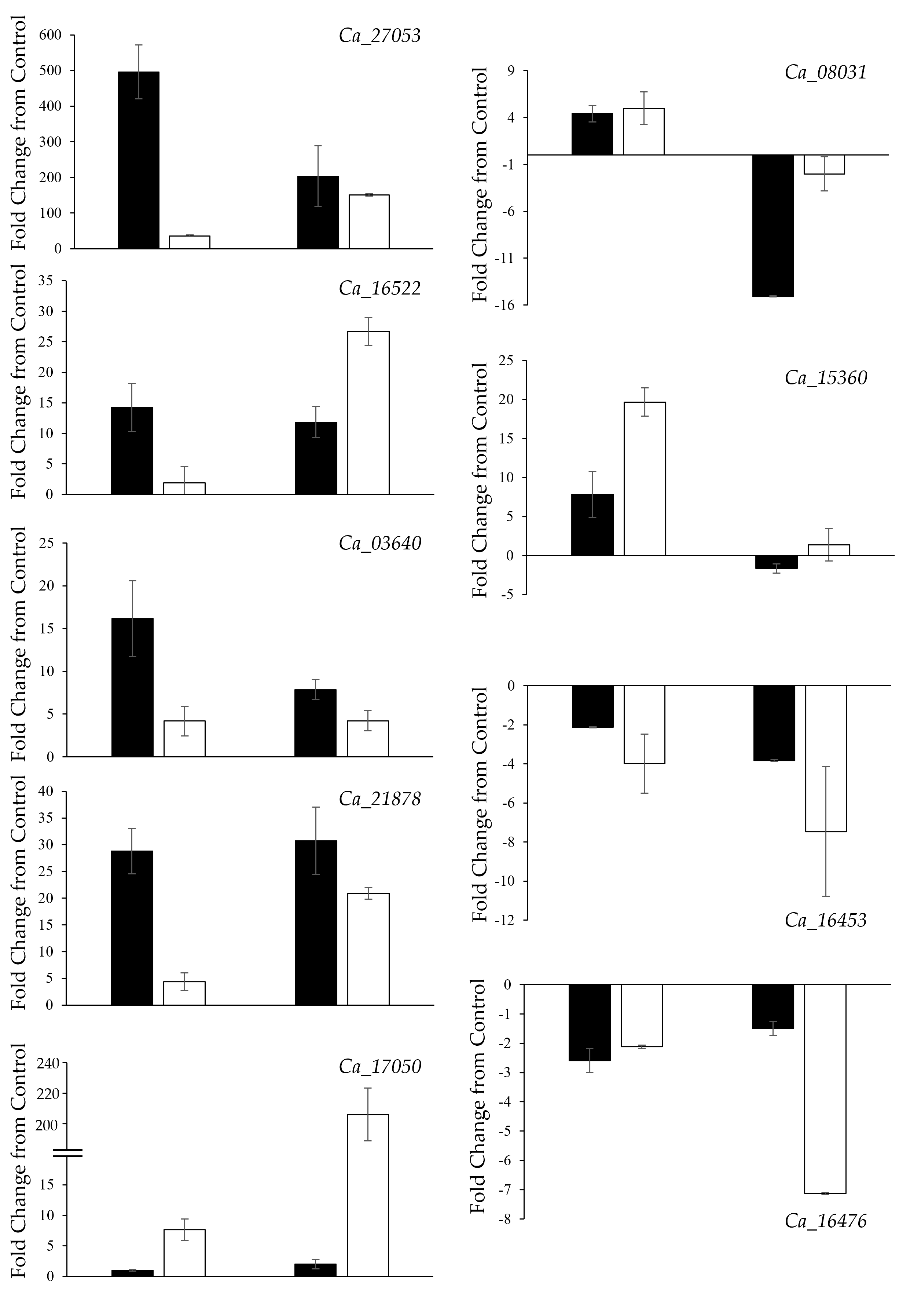

Supplement: Supplementary file 1 [file microorganisms-10-00343-s001.zip › Supplementals/Figure S1.png]
